# Ethiopian progress towards achieving the global nutrition targets of 2025: analysis of sub-national trends and progress inequalities

**DOI:** 10.1186/s13104-020-05408-4

**Published:** 2020-12-09

**Authors:** Nebyu Daniel Amaha

**Affiliations:** Department of Nutrition and Dietetics, College of Health Sciences, Mekelle University, P.O Box: 1871, Mekele, Tigray Ethiopia

**Keywords:** Average annual reduction rate, AARR, Malnutrition, Undernutrition, Stunting, Wasting, Global nutrition targets, Ethiopia

## Abstract

**Objectives:**

The World Health Assembly (WHA) developed six global nutrition targets that focus on child and maternal nutrition. The progress made by individual countries is available as a yearly global nutrition report. However, reporting the national progress might mask important sub-national differences. This study aimed to measure the progress of the 11 regions of Ethiopia towards achieving the 2025 WHA targets using average annual reduction rates (AARR).

**Results:**

Ethiopia is off-track in meeting the five global nutrition targets studied. The national AARR of stunting is 2.3 against a target of 5.3, for wasting the current AARR is 3.1 against a target of 5.3. The AARR of non-exclusive breastfeeding was 2.1 close to the target of 2.7. Anemia in women of reproductive age increased across all the regions of Ethiopia. The majority of Ethiopian regions were on track to achieving the overweight and exclusive breastfeeding targets by 2025. There is an urgent need to address anemia in Ethiopian women of reproductive age because its prevalence has been increasing. Secondly, the progress to reduce wasting and stunting in children under-5 has not been enough and more work needs to be done if Ethiopia is to achieve the 2025 targets.

## Introduction

Malnutrition is a preventable cause of death in almost half of children under 5 in developing countries [1]. The importance of nutrition for development has been recognized by governments and other stakeholders. The second goal of the Sustainable Development Goals (SDGs) focuses on nutrition and aims to end hunger, achieve food security, and improve nutrition. To identify opportunities for action and acknowledge efforts, it is important to monitoring the progress a country makes towards achieving the SDG targets [2–4]. In 2012 the World Health Assembly (WHA) endorsed six global nutrition targets to be achieved between 2012 and 2025. These goals focus on maternal, infant, and young child nutrition and included the following six targets which requires countries to reduce the number of stunted children under 5 by 40%; low birth weight by 30%; anemia in women of reproductive age by 50%; maintain wasting to less than 5%; prevent increase in childhood overweight and increase exclusively breastfed infants to at least 50% [5].

Many countries have made commitments towards achieving the 2025 Global Nutrition Goals and Ethiopia is one of these countries. The reports coming out of the Global Tracking Tool website show that Ethiopia is off-track in meeting five of the goals and there is inadequate data to measure the progress made in low birth weight. The prevalence of stunting and wasting in Ethiopia is above the average for a developing country [6]. The 2018 Global Nutrition Report included reports from 194 countries and only 79 countries were on track to achieve at least one target. Out of these 79 countries, 44 were on track to meet one target, and 35 on track to meet two. No country was on track to achieve to the anemia target [7, 8].

The progress a country makes in achieving the 2025 is available as the annual Global Nutrition Report and online here [6]. This report is calculated at national level and indicates the progress a country has made. The prevalence and/or progress of reducing malnutrition is not uniformly distributed across the Ethiopian regions. For example, the prevalence of stunting varies from 13.9% in Addis Ababa the capital to 48.7% in Tigray region. The national stunting prevalence of 36.8%, thus, does mask the subnational prevalence distribution. Similarly, reporting the progress made in reducing the levels of malnutrition at national level only hides important progress variations. Therefore, this study first aimed to measure the subnational (regional) progress towards the WHA nutrition goals; and secondly to identify the required AARR targets needed to achieve the 2025 targets.

## Main text

### Methodology

The progress towards the global nutrition targets is classified into three categories based on the WHO-UNICEF Technical Expert Advisory Group on Nutrition Monitoring (TEAM) recommendations. This classification compares a country’s required AARR to achieve the target with its current AARR. If the current AARR is greater than the required AARR, the progress is on track; otherwise, it is off track. Moreover, progress has further been classified into off track with some progress and off track with no progress or worsening (Table 1). The acceptable prevalence level for stunting, wasting, and low birth weight is 5% and a country is considered on track if the national prevalence is below 5% [2].

**Table 1:** Rules of interpreting the progress made towards the global nutrition goals based on average annual reduction rate (AARR) [1]

Indicator	On track	Off-track—some progress	Off-track—no progress worsening
Stunting	AARR ≥ 5.3	5.3 > AARR ≥ 0.5	AARR < 5.3 and AARR < 0.5
Anemia	AARR ≥ 5.2	5.2 > AARR ≥ 0.5	AARR < 0.5
NEBF	AARR ≥ 2.74	2.74 > AARR ≥ 0.8	AARR < 0.8
Wasting	Level < 5%	Level ≥ 5% but AARR ≥ 2.0	Level ≥ 5% and AARR < 2.0
Overweight	AARR ≥ − 1.5	AARR < − 1.5	

*NEBF* non-exclusive breastfeeding, *NEBF* 100-exclusive breastfeeding

#### Data source

Data from the publicly available reports of the nationally-representative demographic health surveys (DHS) available after 2011 were used [9–12]. Because the 2014 and 2019 mini-DHS did not report subnational breastfeeding prevalence, two data points from 2011 and 2016 were used. Although the baseline year for the global targets is 2012, the country estimates anywhere between 2005 and 2012 could be used [13]. Therefore, the 2011 DHS survey was used as a baseline year in this study to track the progress toward 2025 goals. There were no sub-national estimates for anemia of women aged 15–49 years in 2014 and 2019, and thus the 2011 and 2016 DHS were used. According to the 2016 EDHS, only 13% of Ethiopian children were weighed at birth [11] and because there is no enough data to make accurate tracking measurements and thus the tracking for low birth weight has not been undertaken in this study (see Additional file 1).

Monitoring the prevalence of not exclusively breastfeed (NEBF) children to track target 5 is recommended as it makes it easier to see the AARR rate and measure progress [2] NEBF is given by 1—EBF, and thus the global goal would be to reduce NEBF from 62% (2012 global estimate) to 50% in 2025. Furthermore, TEAM proposed that 30% or less would be an acceptable prevalence of NEBF level [2].

#### Formulae and data analysis

The AARR is given by the following formula [13, 14] AARR = 1 − EXP (β); where (β) is the coefficient of the linear regression equation of prevalence and year. To calculate β, first the logarithm of the prevalence is calculated, then this log value is regressed with a year and β calculated as the coefficient of the equation. Finally, the exponential logarithm β of is taken and subtracted from 1. When there are only two prevalence estimate points, the formula for AARR is simplified to the logarithm of the ratio of the two prevalence [2]; AARR = ln (P_t_/P_t + n_)/n; where P_t_ is the starting prevalence (baseline), P_t + n_ is the final prevalence, and n is the number of years between the two data points. All statistical analyses were carried out using the R Studio version 3.4.2.

### Results

The national level of stunting has shown a downward trend decreasing from 44.4% in 2011 to 36.8% in 2019 (Table 2). The highest decrease in the prevalence of stunting was in Addis Ababa; whereas, stunting increased in Harari region between 2011 and 2019. The prevalence of wasting increased from its baseline of 10.3% in 2011 to 9.2% in 2019. Tigray region showed the highest percentage of wasted children, where 21% of children under-5 were wasted in 2019. The prevalence of overweight children under-five has increased from 1.8% in 2011 to 2.1% in 2019 (Table 2). The capital Addis Ababa has the highest percentage of overweight children under-5. The national prevalence of anemic women aged 15–49 increased from 16.6% in 2011 to 23.6% in 2016 and the increase was across all the regions (Table 2).

**Table 2:** Percent prevalence of stunting, wasting, and overweight of children under 5, non-exclusive breastfed (NEBF) children under 6 months, and anemia in women of reproductive age in Ethiopia according to four national surveys [9–12]

Region	Stunting		Wasting		Overweight		Anemia		NEBF	
	2011	2025^a^	2011	2019^b^	2011	2019^c^	2011	2025^a^	2011	2025^a^
Addis Ababa	21.84	13.10	9.7	7.2	5.65	4.50	9.3	4.65	64.3	45.0
Affar	50.39	30.24	4.6	2.3	2.14	1.60	34.8	17.4	81.0	56.7
Amhara	51.62	30.97	19.5	13.9	2.08	0.80	16.6	8.3	28.4	19.8
Ben Gumuz	49.13	29.48	9.9	7.6	1.73	1.60	19.1	9.55	55.4	38.8
Dire Dawa	36.53	21.92	9.9	6.1	2.13	1.60	28.8	14.4	60.0	42.0
Gambela	27.36	16.42	12.3	5.8	0.69	0.50	19.4	9.7	70.5	49.3
Harari	30.34	18.20	12.5	12.5	2.02	2.50	19.4	9.7	56.8	39.8
Oromiya	41.46	24.87	9.1	4.2	1.54	3.20	19.2	9.6	53.4	37.4
SNNP	44.30	26.58	9.7	4.7	2.25	1.40	11.3	5.65	47.2	33.0
Somali	33.13	19.88	7.6	6.3	1.40	0.80	44	22	81.0	56.7
Tigray	51.49	30.90	22.2	21.1	0.99	1.90	12.4	6.2	41.4	28.9
National	44.44	26.66	10.3	9.2	1.8	2.1	16.6	8.3	48.0	33.6

*SNNP* South Nations Nationalities and People’s region, *Ben Gumuz* Benishangul Gumuz region

^a^Required prevalence to achieve the 2025 target

^b^Target is to keep prevalence below 5%

^c^To have no increase in the prevalence of overweight children

Nationally, Ethiopia is off track in the five out of the six WHA’s global nutrition goals (Table 3). The AARR shows that there has been some progress in reducing the stunting, wasting, and non-exclusive breastfeeding levels; whereas, there has been no progress in reducing levels of anemia and overweight. Addis Ababa, with 6.4 AARR of stunting, is the only administrative city which is on track to meet the 2025 goal of reducing stunting levels by 40%. Gambela and Somali regions are the two regions that did not make any progress in reducing wasting levels. Eight out of the eleven regions in Ethiopia are on track of meeting the overweight targets. Although Ethiopia is off track in meeting the NEBF rate, nine out of the 11 regions are on track of reducing it by 30% (Table 3).

**Table 3:** The average annual reduction rate (AARR) of stunting, wasting, overweight of children under 5 years old, AARR of anemia in reproductive age women, and AARR of non-exclusive breastfeeding (NEBF) of infants under 6 months in Ethiopian regions

Region	Stunting (≥ 5.3)^a^	Wasting	Overweight (≥ − 1.5)^a^	Anemia (≥ 5.2)^a^	NEBF (≥ 2.74)^a^
Addis Ababa	6.43	3.1	1.77	4.65	7.6
Affar	2.18	7.5	6.96	17.4	11.6
Amhara	2.31	4.8	5.63	8.3	− 6.3
Ben Gumuz	2.01	2.9	4.98	9.55	10.7
Dire Dawa	3.05	6.5	4.94	14.4	10.5
Gambela	4.92	9.0	4.47	9.7	8.2
Harari	− 2.06	0.2	− 1.60	9.7	− 0.6
Oromiya	1.91	6.6	− 9.98	9.6	3.3
SNNP	2.73	7.1	6.57	5.65	0.9
Somali	1.76	2.5	6.50	22	5.4
Tigray	1.10	1.2	− 6.41	6.2	8.4
National	2.35	2.1	− 2.16	8.3	2.0

*Ben Gumuz* Benishagul Gumuz region, *SNNP* South Nations Nationalities and People’s region

^a^Required AARR to achieve the 2025 goal

### Discussion

This study aimed to measure how the different Ethiopian regions were progressing towards achieving the global nutrition goals of 2025. No progress has been made in reducing anemia and overweight prevalence. The national level of stunting levels has been decreasing at AARR of 2.35 (Table 2). The current national prevalence of stunting is 38.6% and if Ethiopia is to achieve the 2025 target, it needs to reduce it to 26.6% (Table 2) which requires an AARR of 6% [15]. Addis Ababa is the only region on track towards achieving the stunting target of 2025 (Table 3). Addis Ababa is the economic and political center of Ethiopia and the number of stunted children is decreasing at a rate of 6.3% per year, considerably higher than the national AARR 2.35 (Table 3). The number of overweight children is increasing faster in Africa and Asia than the rest of the world [16]. Nationally, the number of overweight children is increasing in three Ethiopian regions of Harari, Oromiya, and Tigray while it has not increased in the rest of the regions (Table 2). The national AARR of overweight is − 2.6 and if this rate continues until 2025, the number of overweight children would increase in 2025. The Ethiopian national increase of overweight is 2.6% which is higher than the AARI of 2% in India and lower than the 7% in Nepal [17].

Wasting in children under 2 years old increases the risk of overweight later in life and breastfeeding reduces the risk [16]. This indicates that the nutrition goals are interlinked making a progress would help in other targets. The national AARR of wasting is 3.1 which indicates that some progress is being made. The 2019 country profile report did not include the 2019 Mini EDHS report and thus the national reduction rate was off track with no progress made; however, when data from 2019 was included it shows that most of the Ethiopian regions are making some progress. Wasting is associated with double the mortality risk than stunting [18] and since both share causal factors addressing one would help the other.

Only 31 countries out of 194 were on track for achieving the exclusive breastfeeding target in 2018 [19]. In 2019, 59% of Ethiopian infants under 6 months were breastfed [12]. Although the global 50% target of exclusively breastfed infants has been achieved, the TEAM target of 70% of exclusive breastfed or 30% or less of non-exclusively breastfed is yet to be achieved. This goal requires a 2.74% annual AARR and the national AARR is 2.0; except Harari and Amhara regions, all the other regions of Ethiopia are on track to achieve this target (Table 3). If Ethiopia is to achieve the 2025 reducing anemia by 50% it needs to reduce its prevalence down to 8.3% in 2025 (Table 2). Preventing and treating anemia in women of reproductive age is beneficial to the pregnancy outcomes for mothers and infants, children's school performance, and women's work productivity. Moreover, controlling anemia in pregnant women is one way of preventing low birth weight and reducing perinatal and maternal mortality [20]. There has been no progress towards reducing anemia levels and the gap between the required and the current is highest in anemia reduction rate (Table 3).

### Conclusion

The results of this study show that Ethiopia is off-track in meeting the WHA Global Nutrition Targets. Nationally, there has been some progress made in reducing stunting, wasting and exclusive breastfeeding targets whereas there was no progress made in achieving the overweight and anemia targets. The subnational analysis reveals that the capital Addis Ababa is the only region on track to achieve 40% reduction of stunting by 2025. Moreover, most of the regions are on track to achieve the exclusive breastfeeding by 2025. Gambela and Somali regions are not making any progress in reducing wasting levels and there hasn’t been any progress made in reducing anemia levels as a whole. The national anemia levels in women of reproductive age need an urgent attention. Despite making some progress no Ethiopian region is on track to reduce achieve the wasting target.

## Limitations

This study has some limitations. Firstly, the more data points are available between the baseline and the current rate the better the predictions. However, nationally representative data for anemia and exclusive breastfeeding were available for two data points in 2011 and 2016 and this might not reflect the current situation and the interpretation of these two reduction rates must consider this. Secondly, this study assumes that the current reduction rates do not change and the predictions whether the targets would be achieved are based on this assumption.

## Supplementary information

**Additional file 1.** This is an excel table showing the data used in this study and its analysis.

## Data Availability

The data used in this study are available in Additional file 1.

## Abbreviations

AARR: Average Annual Reduction Rate
DHS: Demographic Health Survey
EDHS: Ethiopian Demographic Health Survey
EBF: Exclusive breastfeeding
NEBF: Non-exclusive breastfeeding
WHA: World Health Assembly
SDG: Sustainable Development Goals
TEAM: UNICEF Technical Expert Advisory Group on Nutrition Monitoring
